# Excess mortality from breast cancer 20 years after diagnosis when life expectancy is normal

**DOI:** 10.1054/bjoc.2000.1616

**Published:** 2001-03

**Authors:** W J Louwman, W J Klokman, J W W Coebergh

**Affiliations:** Eindhoven Cancer Registry, Comprehensive Cancer Centre South (IKZ), PO Box 231, Eindhoven, 5600 AE; Department of Public Health, Erasmus University Rotterdam, PO Box 1738, Rotterdam, 3000 DR; Department of Epidemiology, The Netherlands Cancer Institute, Plesmanlaan 121, Amsterdam, 1066 CX, The Netherlands

**Keywords:** breast cancer, long-term survival, causes of death

## Abstract

In a population-based study, causes of death were traced of 418 deceased breast cancer patients diagnosed in 1960–1979 who survived at least 10 years after diagnosis. The pattern of causes of death in these patients was compared with the general female population using standardized mortality ratios (SMRs). Of 418 patients surviving at least 10 years, 196 (47%) died from breast cancer and 50 (12%) died from another cancer. The SMR for breast cancer was 15.8 (95% CI: 13.1–18.8) 10–14 years after diagnosis; it was still 4.7 (95% CI: 2.6–7.8) after 20 years. Overall mortality was higher than expected 10–14 years after diagnosis (SMR: 1.3; 95% CI: 1.1–1.5), but lower after more than 20 years (SMR: 0.6; 95% CI: 0.4–0.7). Despite a normal (or even improved) life expectancy for breast cancer patients 20 years after diagnosis the risk of dying from this disease remained elevated. © 2001 Cancer Research Campaign http://www.bjcancer.com


*British Journal of Cancer* (2001) **84**(5), 700–703
© 2001 Cancer Research Campaign
doi: 10.1054/ bjoc.2000.1616, available online at http://www.idealibrary.com on IDEAL

http://www.bjcancer.com

### Excess mortality from breast cancer 20 years after diagnosis when life expectancy is normal

**WJ Louwman[1,2], WJ Klokman[3] and JWW Coebergh[1,2]**

[1]Eindhoven Cancer Registry, Comprehensive Cancer Centre South (IKZ), PO Box 231, 5600 AE Eindhoven; [2]Department of Public Health, Erasmus University Rotterdam, PO Box 1738, 3000 DR Rotterdam; [3]Department of Epidemiology, The Netherlands Cancer Institute, Plesmanlaan 121, 1066 CX Amsterdam, The Netherlands

**Summary** In a population-based study, causes of death were traced of 418 deceased breast cancer patients diagnosed in 1960–1979 who survived at least 10 years after diagnosis. The pattern of causes of death in these patients was compared with the general female population using standardized mortality ratios (SMRs). Of 418 patients surviving at least 10 years, 196 (47%) died from breast cancer and 50 (12%) died from another cancer. The SMR for breast cancer was 15.8 (95% CI: 13.1–18.8) 10–14 years after diagnosis; it was still 4.7 (95% CI: 2.6–7.8) after 20 years. Overall mortality was higher than expected 10–14 years after diagnosis (SMR: 1.3; 95% CI: 1.1–1.5), but lower after more than 20 years (SMR: 0.6; 95% CI: 0.4–0.7). Despite a normal (or even improved) life expectancy for breast cancer patients 20 years after diagnosis the risk of dying from this disease remained elevated. © 2001 Cancer Research Campaign http://www.bjcancer.com

**Keywords:** breast cancer; long-term survival; causes of death

Prognosis for breast cancer patients has improved over the past few decades; relative 10-year survival rates increased from 47% for patients diagnosed in 1970–1974 to 61% for patients diagnosed in 1980–1984 (Nab et al, 1994a). Studies in the UK, Sweden, New Zealand, and The Netherlands found that about 20 years after diagnosis the life expectancy of patients with breast cancer resembles that of the general population (Brinkley and Haybittle, 1975; Hibberd et al, 1983; Rutqvist and Wallgren, 1985; Fentiman et al, 1994; Nab et al, 1994b), suggesting that patients can then be considered cured. However, breast cancer recurrences or metastases may develop decades after initial diagnosis and treatment (Hibberd et al, 1983; Nab et al, 1994b) and a higher death rate due to breast cancer has been reported up to 40 years after diagnosis (Rutqvist and Wallgren, 1985).

Since the age-adjusted incidence rate in Southeast Netherlands, as in many other Western countries, has doubled since 1960 (Nab et al, 1993) and prognosis has improved, the number of long-term survivors of breast cancer has increased markedly. However, it remains unclear whether these patients should still be considered at increased risk of being affected by breast cancer, even though this is important for follow-up surveillance and the natural history of breast cancer.

In the present population-based study, we investigated mortality of breast cancer patients who had survived at least 10 years after diagnosis, comparing this with that of the general population by calculating standardized mortality ratios (SMRs).

*Received 18 September 2000*
*Revised 9 November 2000*
*Accepted 10 November 2000*

*Correspondence to:* WJ Louwman

### METHODS

Data on diagnosis, stage of disease, and treatment were obtained from the population-based Eindhoven Cancer Registry, which has collected data on new cancer patients since 1955 according to international guidelines (Parkin et al, 1997). The registry covers an area of 2500 $km^2$ in Southeast Netherlands with a population of almost one million inhabitants. The data on first and second primary breast tumours were obtained, after notification by pathologists, from pathology reports, hospital records, and the regional Radiotherapy Institute. Access to specialized care was good during the whole period.

Medical registration of causes of death in the Netherlands is similar to that in many other countries, according to WHO guidelines. The attending physician must report the underlying cause of death. Statistics Netherlands assigns a code for the underlying cause of death according to the International Classification of Diseases, ninth revision (ICD-9) (World Health Organisation, 1977). If any other medically relevant information is mentioned on the death certificate, the secondary cause of death is also recorded. The anonymous cause-of-death register, maintained by Statistics Netherlands, is listed per municipality according to sex and age (derived from date of birth and date of death).

Patients were tracked by the Central Bureau of Genealogy in order to obtain the unique death certificate number. Statistics Netherlands was provided with a list, according to municipality of death, the date of birth, date of death and death certificate number (when available). The data were obtained at an aggregate level fit for data analysis.

All 1460 patients diagnosed with breast cancer in 1960 through 1979 who survived at least 10 years after initial diagnosis were selected for the present study. Follow-up was completed until 1.1.1994, when 487 long-term survivors had died. The causes of death of those patients who survived at least 10 years but died in

**Table 1** Characteristics of 418 deceased breast cancer patients diagnosed in 1960–1979 who survived at least 10 years

| | Period of diagnosis | | |
|---|---|---|---|
| | **1960–1969 *n* (%)** | **1970–1979 *n* (%)** | **Total *n* (%)** |
| Age at diagnosis (yrs) | | | |
| <40 | 6 (7.8) | 16 (4.7) | 22 (5.3) |
| 40–49 | 21 (27) | 58 (17) | 79 (19) |
| 50–59 | 21 (27) | 83 (24) | 104 (25) |
| 60–69 | 21 (27) | 117 (34) | 138 (33) |
| ≥ 70 | 8 (10) | 67 (20) | 75 (18) |
| Tumour stage | | | |
| Local | 42 (55) | 168 (49) | 210 (50) |
| Regional | 34 (44) | 121 (36) | 155 (37) |
| Distant | 0 (0) | 4 (1.2) | 4 (1.0) |
| Unknown | 1 (1.3) | 48 (14) | 49 (12) |
| Histological type | | | |
| Ductal | 49 (64) | 300 (88) | 349 (84) |
| Lobular | 2 (2.6) | 12 (3.5) | 14 (3.3) |
| Mucinous | 1 (1.3) | 9 (2.6) | 10 (2.4) |
| Medullar | 2 (2.6) | 6 (1.8) | 8 (1.9) |
| Sarcoma | 1 (1.3) | 0 (0) | 1 (0.2) |
| Not otherwise specified | 22 (29) | 14 (4.1) | 36 (8.6) |
| Radiotherapy | | | |
| No | 17 (22) | 144 (42) | 161 (39) |
| Yes | 60 (78) | 197 (58) | 257 (61) |
| Total (% of study population) | 77 (13) | 341 (87) | 418 (100) |

1970–1979 (*n* = 47) were not traced, because of the high search costs per year and the low yield. For 22 patients the cause of death could not be traced. Consequently, observed causes of death are presented only for 418 patients.

In the analysis, intervals of follow-up (10–14, 15–19 and 20 years or more) were distinguished. Causes of death were grouped together according to major disease categories. SMRs were calculated for comparison of causes of death in the study population with the general female population. In this person-years type of analysis, the ratio of the observed (O) and expected (E) numbers of deaths was determined. Time at risk began at the date of diagnosis and ended at the date of death or the end of the follow-up period (1.1.1994), whichever occurred first. Taking into account the person-years of observation in the cohort (by age and calendar period), expected numbers of death were computed using age-, and calendar-period-specific cancer death rates from the area of the Eindhoven Cancer Registry. The confidence limits of SMR were obtained with the use of the Poisson distribution of observed numbers (Pearson and Hartley, 1976). SMRs were calculated for all patients together and separately by follow-up interval.

### RESULTS

Clinical characteristics are presented in Table 1 according to period of diagnosis. Adjuvant primary radiotherapy was administered to 53% of patients with localized tumours and 84% of those with regional or distant disease. Only 11 patients received adjuvant systemic therapy, 9 of whom had regional or distant disease at the time of diagnosis, one had a T4 tumour and in one patient tumour stage was unknown.

Of all 418 patients surviving for at least 10 years after diagnosis 196 (47%) died from breast cancer and 50 (12%) from another cancer (Table 2). In another 13 cases breast cancer was recorded as secondary cause of death.

Among those who survived 10–14 years after initial diagnosis the observed number of total deaths was higher than expected (SMR: 1.3; 95% CI: 1.1–1.5) (Table 3). The risk of dying from breast cancer was almost 16 times higher than expected (13.1–18.8). In the patients who survived 15–19 years the SMR for all causes of death was 1.0, with an excess risk of dying from breast cancer (SMR: 11.0; 95% CI: 8.3–14.3). For those patients surviving more than 20 years the observed number of total deaths was lower than expected (SMR: 0.6; 95% CI: 0.4–0.7), but the risk of dying from breast cancer was still almost 5 times higher than expected (95% CI: 2.6–7.8).

The distribution of patients dying from cancer other than breast cancer is shown in Table 4. High, but not statistically significant, SMRs were observed for small bowel and brain tumours and low SMRs for cancer of the stomach, larynx and lung. Another cancer

**Table 2** Causes of death in breast cancer patients surviving for more than 10 years after diagnosis in 1960–1979 for different intervals of follow-up

| | **10–14 yrs *n* (%)** | **15–19 yrs *n* (%)** | **≥20 yrs *n* (%)** | **Total *n* (%)** |
|---|---|---|---|---|
| Cause of death | | | | |
| Breast cancer | 126 (54) | 56 (42) | 14 (27) | 196 (47) |
| Other cancer | 23 (9.7) | 17 (13) | 10 (20) | 50 (12) |
| Cardiovascular disease | 53 (22) | 36 (27) | 17 (33) | 106 (25) |
| Respiratory disease | 7 (3.0) | 3 (2.2) | 4 (7.8) | 14 (3.3) |
| Disease of the digestive system | 4 (1.7) | 3 (2.2) | 3 (1.3) | 10 (2.4) |
| External causes[a] | 6 (2.5) | 4 (3.1) | – | 10 (2.4) |
| Other causes | 16 (6.8) | 13 (10) | 3 (1.3) | 32 (7.6) |
| Total (% of study population) | 235 (56) | 132 (32) | 51 (12) | 418 (100) |

[a]Includes accidents, murder, suicide.

© 2001 Cancer Research Campaign

**Table 3** Number of observed (O) and expected (E) causes of death, standardized mortality ratios (SMRs) and 95% CI in breast cancer patients for different intervals of follow-up

| | 10–14 years | | | | 15–19 years | | | | ≥ 20 years | | | |
|---|---|---|---|---|---|---|---|---|---|---|---|---|
| | O | E | SMR | (95% CI) | O | E | SMR | (95% CI) | O | E | SMR | (95% CI) |
| Cause of death | | | | | | | | | | | | |
| Breast cancer | 126 | 8.0 | 15.8 | (13.1–18.8) | 56 | 5.1 | 11.0 | (8.3–14.3) | 14 | 3.0 | 4.7 | (2.6–7.8) |
| Other cancer | 23 | 32.2 | 0.7 | (0.5–1.1) | 17 | 21.6 | 0.8 | (0.5–1.3) | 10 | 13.4 | 0.7 | (0.4–1.4) |
| Cardiovascular disease | 53 | 89.2 | 0.6 | (0.4–0.8) | 36 | 64.6 | 0.6 | (0.4–0.8) | 17 | 44.2 | 0.4 | (0.2–0.6) |
| Respiratory disease | 7 | 11.7 | 0.6 | (0.2–1.2) | 3 | 9.7 | 0.3 | (0.1–0.9) | 4 | 7.3 | 0.5 | (0.1–1.4) |
| Other causes | 26 | 39.5 | 0.7 | (0.4–1.0) | 20 | 32.2 | 0.6 | (0.4–1.0) | 6 | 24.0 | 0.3 | (0.1–0.5) |
| Total | 235 | 180.7 | 1.3 | (1.1–1.5) | 132 | 133.1 | 1.0 | (0.8–1.2) | 51 | 91.9 | 0.6 | (0.4–0.7) |

was the secondary cause of death for another 9 patients, of whom 4 had female genital cancer.

Patients who died from breast cancer after 10 years were generally younger at diagnosis and at death than those who died from other causes (Table 5). Among patients with localized disease the proportion who died from breast cancer equalized to the proportion dying from other causes, until 20 years after diagnosis. Patients diagnosed with regional disease were more likely to die from breast cancer 10–14 years after diagnosis, whereas the proportion dying from other causes became higher thereafter.

A second primary breast tumour was present in 131 of the 1460 patients (9%). For 76 women with a second breast tumour the cause of death was unknown because the patients were still alive at 1.1.94 ($n$ = 67), died between 1970 and 1979 ($n$ = 5), or the cause of death could not be traced ($n$ = 4). For 55 patients with a second breast tumour the cause of death was known: 39 (71%) died from breast cancer and 16 died from other causes.

### DISCUSSION

Breast cancer patients who have survived 20 years or more after diagnosis and are presumed to have a normal life expectancy were still at a 5-fold increased risk of dying from breast cancer. Mortality from other causes was lower.

**Table 4** Number observed (O) and expected (E), standardized mortality ratios (SMR), and 95% CI for breast cancer patients who survived ≥ 10 years and died from cancer other than breast cancer

| | O | E | SMR | (95% CI) |
|---|---|---|---|---|
| Tumour site (ICD-codes) | | | | |
| Head, Neck, Oesophagus (140–150) | 1 | 1.6 | 0.6 | (0.0–3.5) |
| Stomach (151) | 3 | 7.3 | 0.4 | (0.1–1.2) |
| Small bowel (152) | 2 | 0.3 | 6.1 | (0.8–24) |
| Colon, Rectum (153, 154) | 10 | 14.2 | 0.7 | (0.3–1.3) |
| Liver, Biliary tract, Pancreas (155–157) | 5 | 8.2 | 0.6 | (0.2–1.4) |
| Larynx, Lung (161, 162) | 2 | 5.2 | 0.4 | (0.0–1.4) |
| Cervix uteri (180) | 1 | 1.2 | 0.8 | (0.0–4.6) |
| Corpus uteri (182) | 1 | 1.6 | 0.6 | (0.0–3.5) |
| Ovary (183) | 6 | 4.6 | 1.3 | (0.5–2.8) |
| Urinary tract (188, 189) | 4 | 4.2 | 0.9 | (0.3–2.4) |
| Brain (191) | 2 | 0.7 | 2.7 | (0.3–10) |
| Haemopoietic (200–208) | 6 | 6.7 | 0.9 | (0.3–2.0) |
| Other malignancies | 7 | 11.4 | 0.6 | (0.2–1.3) |
| Total | 50 | 67.3 | 0.7 | (0.6–1.0) |

Registration of cause of death in the Netherlands is similar to that in other countries, according to WHO guidelines. Could over-reporting of breast cancer as the underlying cause of death explain the excess death risk? We think that rather this tendency, if present, declines with the duration of follow-up. Breast cancer would tend only to be reported if there was clear progression in the disease or a second breast cancer had occurred. We found that in 39 (71%) of the 55 patients who had developed a second primary breast tumour, the death was attributed to breast cancer. Fentiman et al (1994) found that 10% of patients surviving more than 20 years since diagnosis of the first tumour died from metastases from contralateral cancer and late deaths from breast cancer were due to new tumours rather than a late manifestation of slow-growing metastases. Of the 51 such patients in our study, 11 (22%) developed a second breast tumour, 7 of whom died from the disease. A total of 14 long-term survivors died from breast cancer (Table 2). So if the 7 deaths in those with a second primary tumour were caused by this second tumour, the other 7 deaths due to breast cancer (50%) resulted from progression of the initial disease.

Irrespective of whether late death from breast cancer was due to metastasis from the initial tumour or from a second primary, the question remains whether excess death from breast cancer can be prevented by longer regular surveillance or better treatment. Routine follow-up by physical examination and an annual or biannual mammography would be sufficient to detect recurrences or a second primary tumour (Rutgers et al, 1989). More importantly, overall mortality in patients who survived over 20 years after initial diagnosis became lower than in the general population. Even if those deceased patients of whom the cause of death was not traced ($n$ = 69) were added to the observed number of deaths the SMRs for overall mortality remained roughly the same (SMR: 1.5, 1.1, and 0.7 for those surviving 10–14 years, 15–19, and ≥ 20 years, respectively).

Patients diagnosed before the age of 50 were more likely to die from breast cancer than from other causes. Proportional mortality from breast cancer is clearly related to age at death (Philips et al, 1999). At younger ages women are less likely to die from other causes, so that the proportion dying from breast cancer is relatively high. We took account of this by calculating standardized mortality rates, but breast cancer mortality remained higher than expected. We also checked whether misclassification of cause of death in the oldest elderly may have affected the high death rates from breast cancer. However, we found that the percent of patients who died from breast cancer was actually lower in the 85+ group than in the total study-population.

*© 2001 Cancer Research Campaign*

**Table 5** Age and stage of breast cancer patients who survived at least 10 years after diagnosis at different intervals of follow-up. Patients who died from breast cancer are compared with patients who died from other causes

| | 10–14 yrs | | 15–19 yrs | | ⩾ 20 yrs | |
|---|---|---|---|---|---|---|
| | Breast cancer *n* (%) | Other cause *n* (%) | Breast cancer *n* (%) | Other cause *n* (%) | Breast cancer *n* (%) | Other cause *n* (%) |
| Mean age (y) at diagnosis (SD) | 56 (12) | 65 (10) | 54 (11) | 62 (10) | 50 (11) | 56 (10) |
| Mean age (y) at death (SD) | 69 (12) | 77 (10) | 71 (11) | 79 (10) | 72 (11) | 80 (9) |
| Age at diagnosis (y) | | | | | | |
| <50 | 43 (81) | 10 (19) | 21 (65) | 11 (35) | 6 (38) | 10 (63) |
| 50–69 | 68 (51) | 65 (49) | 31 (41) | 45 (59) | 8 (24) | 25 (76) |
| ≥70 | 15 (31) | 34 (69) | 4 (16) | 20 (84) | 0 | 2 (100) |
| Tumour stage | | | | | | |
| Local | 58 (48) | 63 (52) | 31 (52) | 29 (48) | 9 (31) | 20 (69) |
| Regional | 54 (64) | 30 (36) | 20 (37) | 34 (63) | 4 (24) | 13 (76) |
| Distant | 2 (67) | 1 (33) | 0 | 1 (100) | 0 | 0 |
| Unknown | 12 (44) | 15 (56) | 5 (25) | 12 (75) | 1 (20) | 4 (80) |
| Total | 126 (54) | 109 (46) | 56 (42) | 76 (58) | 14 (27) | 37 (73) |

The pattern of death due to cancer other than breast cancer was not very surprising. The increased observed/expected ratio for small bowel cancer could also be a random finding. Increased risks after breast cancer, regularly reported for cancer of the colon-rectum, ovary, and uterus (Schenker et al, 1984; Ewertz and Mouridsen, 1985; Harvey and Brinton, 1985; Teppo et al, 1985; Rutqvist et al, 1995) are partly explained by a common aetiology or pathogenesis (e.g., hormonal exposure or susceptibility). The decreased risk for tobacco-related cancers in the lung and head and neck area could also be related to the high socio-economic status of breast cancer patients (Schrijvers et al, 1997), greater health awareness could also play a role.

Previously, excess mortality from breast cancer despite a normal life-expectancy was also observed in British women diagnosed between 1947 and 1950 who survived more than 20 years (Brinkley and Haybittle, 1975, 1984). The present study indicates that these findings are still valid for patients diagnosed in later decades (1960–1979). Despite a normal (or even improved) life expectancy for breast cancer patients 20 years after diagnosis, the risk of dying from this disease remained elevated.

### ACKNOWLEDGEMENTS

We thank EJTh Rutgers, Department of Surgical Oncology, The Netherlands Cancer Institute, Amsterdam and JG Ribot, Department of Radiotherapy, Catherina Hospital, Eindhoven for valuable comments. This study was supported by a grant (IKZ 95–1012) from the Dutch Cancer Society.

### REFERENCES

Brinkley D and Haybittle J (1975) The curability of breast cancer. *Lancet* **ii**: 95–96

Brinkley D and Haybittle J (1984) Long-term survival of women with breast cancer. *Lancet* **i**: 1118.

Ewertz M and Mouridsen H (1985) Second cancer following cancer of the female breast in Denmark, 1943–80. *Natl Cancer Inst Monogr* **68**: 325–329

Fentiman I, Cuzick J, Millis R and Hayward J (1994) Which patients are cured of breast cancer? *Br Med J* **289**: 1108–1111

Harvey E and Brinton L (1985) Second cancer following cancer of the breast in Connecticut 1935–82. *Natl Cancer Inst Monogr* **68**: 99–112

Hibberd A, Horwood L and Wells J (1983) Long term prognosis of women with breast cancer in New Zealand: study of survival to 30 years. *Br Med J* **286**: 1777–1779

Nab H, Voogd A, Crommelin M, Kluck H, Heijden L.v.d. and Coebergh J (1993) Breast cancer in Southeast Netherlands, 1960–1989: trends in incidence and mortality. *Eur J Cancer* **29A**: 1557–1560

Nab H, Hop W, Crommelin M, Kluck H and Coebergh J (1994a) Improved prognosis in breast cancer since 1970 in south-east Netherlands. *Br J Cancer* **70**: 285–288

Nab H, Hop W, Crommelin M, Kluck H, Heijden L.v.d. and Coebergh J (1994b) Improved long-term prognosis in breast cancer: survival rates since 1955 in a Dutch cancer registry. *Br Med J* **309**: 83–86

Parkin D, Whelan S, Ferlay J, Raymond L and Young J (1997) *Cancer incidence in five continents,* Vol. VII, No. 143. IARC Scientific publications: Lyon

Pearson E and Hartley H (1976) *Biometrika tables for statisticians* (ed 3). Biometrika Trust: London, United Kingdom

Philips A, Glendon G and Knight J (1999) Putting the risk of breast cancer in perspective. *N Engl J Med* **340**: 141–144

Rutgers E, Slooten E.v. and Kluck H (1989) Follow-up after treatment of primary breast cancer. *Br J Surg* **76**: 187–190

Rutqvist LE and Wallgren A (1985) Long-term survival of 458 young breast cancer patients. *Cancer* **55**: 658–65

Rutqvist L, Johansson H, Signomklao T, Johansson U, Fornander T and Wilking N (1995) Adjuvant tamoxifen therapy for early stage breast cancer and second primary malignancies. Stockholm Breast cancer Study Group. *J Natl Cancer Inst* **87**: 645–651

Schenker J, Levinsky R and Ohel G (1984) Multiple primary malignant neoplasms in breast cancer patients in Israel. *Cancer* **54**: 145–150

Schrijvers C, Coebergh J and Mackenbach J (1997) Socioeconomic status and comorbidity among newly diagnosed cancer patients. *Cancer* **80**: 1482–1488

Teppo L, Pukkala E and Saxen E (1985) Multiple cancer – an epidemiologic exercise in Finland. *J Natl Cancer Inst* **75**: 207–217

World Health Organisation (1977) *International classification of diseases, injuries, and causes of death, ninth revision*. World Health Organisation: Geneva, Switzerland

© 2001 Cancer Research Campaign 

## References

[PDF_00308] Brinkley D., Haybittle J. L. (1984). Long-term survival of women with breast cancer.. Lancet.

[PDF_00307] Brinkley D., Haybrittle J. L. (1975). The curability of breast cancer.. Lancet.

[PDF_00310] Ewertz M., Mouridsen H. T. (1985). Second cancer following cancer of the female breast in Denmark, 1943-80.. Natl Cancer Inst Monogr.

[PDF_00313] Fentiman I. S., Cuzick J., Millis R. R., Hayward J. L. (1984). Which patients are cured of breast cancer?. Br Med J (Clin Res Ed).

[PDF_00315] Harvey E. B., Brinton L. A. (1985). Second cancer following cancer of the breast in Connecticut, 1935-82.. Natl Cancer Inst Monogr.

[PDF_00317] Hibberd A. D., Horwood L. J., Wells J. E. (1983). Long term prognosis of women with breast cancer in New Zealand: study of survival to 30 years.. Br Med J (Clin Res Ed).

[PDF_00323] Nab H. W., Hop W. C., Crommelin M. A., Kluck H. M., Coebergh J. W. (1994). Improved prognosis of breast cancer since 1970 in south-eastern Netherlands.. Br J Cancer.

[PDF_00326] Nab H. W., Hop W. C., Crommelin M. A., Kluck H. M., van der Heijden L. H., Coebergh J. W. (1994). Changes in long term prognosis for breast cancer in a Dutch cancer registry.. BMJ.

[PDF_00320] Nab H. W., Voogd A. C., Crommelin M. A., Kluck H. M., vd Heijden L. H., Coebergh J. W. (1993). Breast cancer in the southeastern Netherlands, 1960-1989: trends in incidence and mortality.. Eur J Cancer.

[PDF_00333] Phillips K. A., Glendon G., Knight J. A. (1999). Putting the risk of breast cancer in perspective.. N Engl J Med.

[PDF_00335] Rutgers E. J., van Slooten E. A., Kluck H. M. (1989). Follow-up after treatment of primary breast cancer.. Br J Surg.

[PDF_00339] Rutqvist L. E., Johansson H., Signomklao T., Johansson U., Fornander T., Wilking N. (1995). Adjuvant tamoxifen therapy for early stage breast cancer and second primary malignancies. Stockholm Breast Cancer Study Group.. J Natl Cancer Inst.

[PDF_00337] Rutqvist L. E., Wallgren A. (1985). Long-term survival of 458 young breast cancer patients.. Cancer.

[PDF_00343] Schenker J. G., Levinsky R., Ohel G. (1984). Multiple primary malignant neoplasms in breast cancer patients in Israel.. Cancer.

[PDF_00345] Schrijvers C. T., Coebergh J. W., Mackenbach J. P. (1997). Socioeconomic status and comorbidity among newly diagnosed cancer patients.. Cancer.

[PDF_00347] Teppo L., Pukkala E., Saxén E. (1985). Multiple cancer--an epidemiologic exercise in Finland.. J Natl Cancer Inst.

